# Time to progression ratio in cancer patients enrolled in early phase clinical trials: time for new guidelines?

**DOI:** 10.1038/s41416-018-0245-0

**Published:** 2018-10-17

**Authors:** Sarah Watson, Jessica Menis, Capucine Baldini, Patricia Martin-Romano, Jean-Marie Michot, Antoine Hollebecque, Jean-Pierre Armand, Christophe Massard, Jean-Charles Soria, Sophie Postel-Vinay, Xavier Paoletti

**Affiliations:** 1 Drug Development Department, Gustave Roussy Cancer Campus, Université Paris-Saclay, Villejuif, France; 2 UMR981, ATIP-Avenir team, INSERM, Villejuif, France; 3 Biostatistics and Epidemiology Department, Gustave Roussy Cancer Campus, Villejuif, France; 4 CESP-OncoStat, INSERM, Paris-Sud University, Villejuif, France

## Abstract

**Background:**

Reliable evaluation of treatment benefit in early phase clinical trials is necessary. The time to progression ratio (TTPr), which compares successive TTP in a single patient, is a powerful criteria for determining targeted or immune therapies efficacy.

**Methods:**

We evaluated 205 TTPr in a large cohort of 177 advanced cancer patients enrolled in at least two Phase 1/1b trials (out of 2827 phase 1/1b-treated patients) at Gustave Roussy.

**Results:**

This first wide description of TTPr showed that, under the hypothesis of overall absence of treatment line effect, the median TTPr was 0.7 and that 25% of patients presented a TTPr above the conventional efficacy threshold of 1.3.

**Conclusions:**

A higher median TTPr and a larger proportion of patients above the 1.3 threshold should therefore be achieved to conclude to drug efficacy. New guidelines for TTPr interpretation and calibration are proposed, which warrant independent prospective validation.

## Background

The early detection of signs of clinical efficacy is a pivotal element to further develop new drugs. Most early phase clinical trials have relied on imaging parameters based on Response Evaluation Criteria in Solid Tumours v1.1 (RECIST), or Cheson criteria for lymphomas to assess efficacy. However, these parameters are heterogeneous across patients; using the patient as its own control has therefore been proposed to allow better detection of clinical benefit.^1–3^ The time to progression ratio (TTPr), which compares the TTP of two consecutives lines of treatment within the same patient, is becoming increasingly used in early phase trials to detect drug benefit.^4–8^ If the treatment is inactive, TTP at line +2 (TTP2) is expected to be shorter than TTP at line +1 (TTP1), whereas a TTP2/TTP1 ratio > 1.3 might reflect treatment benefit.^9^ However, although the above 1.3 threshold is widely used, data are missing to support its choice, and which TTPr should be achieved to reflect treatment benefit is still debatable. We aimed at describing TTPr in a large cohort of patients participating in two or more phase 1/1b studies in Gustave Roussy Drug Development Department (DITEP). This population was chosen to minimise frequent bias in TTPr calculation (as the frequency of disease radiological evaluation was homogeneous within trials), and to work on a representative population of early phase trial patients.

## Methods

All patients with refractory solid tumours or lymphomas included in at least two Phase 1/1b clinical trials between 2009 and 2016 were eligible. TTP at a given treatment line was defined as the time from treatment initiation to the first documented progression. TTPr was the ratio of two successive TTP. In case a patient participated in more than two clinical trials, TTPr was calculated for each sequence of two trials. In patients who did not progress before starting a second phase 1 trial, the corresponding line of treatment was excluded from the primary analyses. As criteria for trial allocation were constant between 2009 and 2016, we hypothesised that the likelihood of receiving the most effective drug during the first or second trial was similar. Accordingly, the null hypothesis was that the median global “treatment effect” would correspond to an “absence of effect”, since 50% of patients would have received the most effective drug in trial 1, and 50% in trial 2.

## Results

Between 2009 and 2016, 2827 patients were included in a Phase 1/1b trial at the DITEP. Among them, 196 patients had been enrolled in more than one Phase1/1b trial, and 177 (90.3%) patients who had uncensored first TTP could be included in the analysis. A total of 205 TTPr corresponding to 387 treatment lines could be calculated and 135/205 (65.9%) TTPr were based on strictly consecutive trials. Main demographics and clinical data are presented in Supplementary Table 1; 56/177 (31.6%) patients were enrolled in at least one trial based on molecular orientation. Patients were enrolled in 101 different trials, which investigated 159 therapeutic agents administered as monotherapy or in combination; 20/101 (19.8%) trials required to have a specific molecular alteration.

Median TTP1, TTP2 and TTP3 were 3.8, 2.5, and 1.8 months, respectively (Supplementary Figure 1A). TTP1 was not associated with the number of previous treatment lines (*p* = 0.44). Median OS was 8.8 months (95% CI = 7.4, 10.1) (Supplementary Figure 1B). Correlation between TTP1 and TTP2 (or TTP2 and TTP3) was moderate, with Spearman rho = 0.25.

**Fig. 1:**
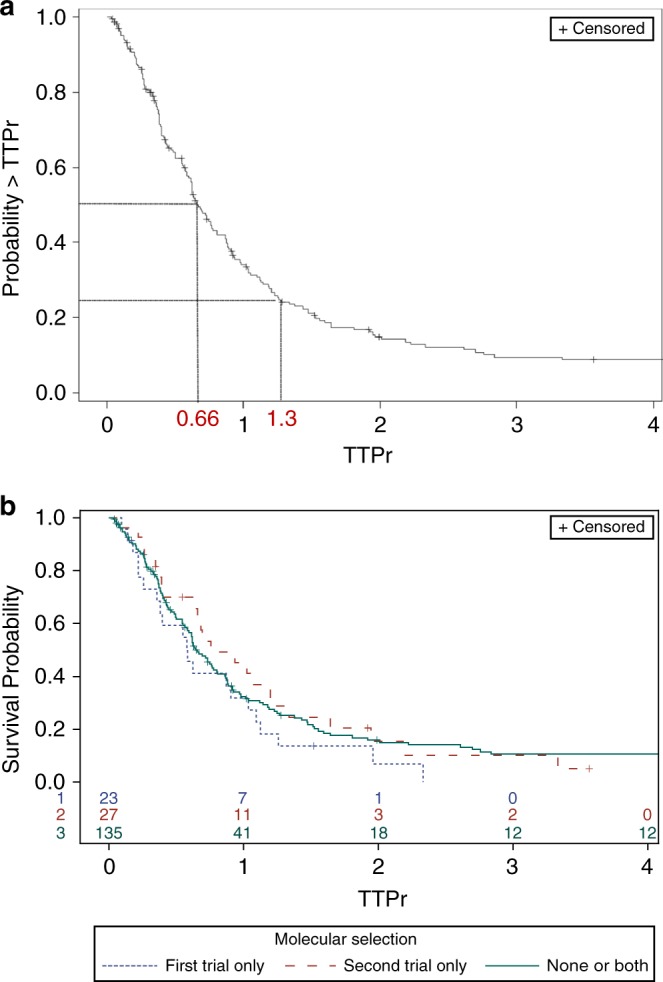
TTPr distribution in patients enrolled in successive Phase1/1b trials. TTPr is reported using Kaplan–Meier estimate to account for possibly censored observations. This estimate is hypothesised to correspond to a reference population with “overall no treatment line effect”. A: Distribution of TTPr in the overall population. The median TTPr in this population is highlighted with dotted lines. B: Distribution of TTPr according to the participation to a trial based on molecular orientation. Data is represented for patients included in a molecularly-oriented trial as first trial only, second trial only, and in none or in both trials

TTPr distribution is depicted in Fig. 1a. Median TTPr was 0.66 (95% CI = 0.6, 0.8). The probability of presenting a TTPr above the standard 1.3 threshold was 24.2% (95% CI = 0.18, 0.30); the probability of presenting a TTPr above 1.55, 2 or 3 was 20%, 15% and 9.5%, respectively. TTPr distribution did not significantly differ whatever the age at diagnosis, the primary tumour location, the Royal Marsden Hospital (RMH) prognostic score, or the number of previous treatment lines. A trend towards better TTPr in patients treated with precision medicine as second trial was observed, although not significant (*p* = 0.17) (Fig. 1b). The results were similar when the analysis was performed after excluding patients treated with immune checkpoint blockers (data not shown). The correlation between TTPr and OS was mild (Spearman rho = 0.22; Supplementary Figure 1C), and inferior to the correlation between TTP2 and OS (Spearman rho = 0.59; Supplementary Figure 1D).

## Discussion

In the original study by Van Hoff, the null hypothesis was that less than 15% of patients treated according to molecular profiling would have TTPr above 1.3. With 27% (18/66) of patients showing TTPr above 1.3, the authors concluded that molecular profiling led to therapeutic benefit.^9^ Improved results have been reported more recently in the prospective MOSCATO-01 study, where 33% of the 199 molecularly selected patients achieved clinical benefit with TTPr > 1.3—suggesting refinements in patient molecular selection, or improved selectivity or potency of investigational compounds.^10^ More stringent thresholds have sometimes been used, although not supported by clinical or statistical data.^4^ Our data show that approximately 25% of patients included in successive early phase studies present TTPr above the standard 1.3 threshold in a situation of overall absence of treatment effect. This supports that a median TTPr above 0.7 and a higher (>25%) proportion of patients above the 1.3 threshold, should be achieved to conclude to drug efficacy in early phase trials. This is particularly true for trials where a molecular enrichment of patients is applied, as a higher benefit can be expected in this situation. The stringency of the above proposed threshold would therefore need to be adjusted on several factors, including the presence of a targetable molecular alteration, the median TTP, and the time per treatment index (TPTi)—the latter reflecting the agressivity of the disease at the patient level.^11^ For example, a TTPr of 1.8 could be targeted in studies with known actionable alteration and in aggressive diseases, whereas a TTPr of 1.3 would be more suited studies without known molecular target or in indolent diseases. An interesting validation for the proposed ratios would consist in prospectively evaluating TTPr in complete datasets from phase 1 trials and correlate the outcomes with the phase 2 and 3 results.

No correlation between TTPr and OS could be evidenced in our series, contrasting with previous reports.^4,9^ Beyond methodological differences, this may be due to some limitations of the present study, including patients heterogeneity—a characteristic of Phase 1 trials population—or the fact that not all patients were included in two strictly consecutive clinical trials, leading to potential decrease in TTP2. Moreover, it is acknowledged that TTPr can be used as endpoint when TTP1 and TTP2 are highly correlated.^12^ This was not the case is our series and supports that stronger effects on TTPr are required to conclude to drug efficacy in Phase 1 trials.

To summarise, this study represents the first description of TTPr distribution in a large cohort of phase 1/1b cancer patients and provides guidelines for using TTPr analysis as outcome of treatment activity. In a context where early evaluation of drug efficacy is becoming increasingly important in phase 1 trials, we believe that our work provides useful bases for implementing the use of TTPr in drug development.

## Electronic supplementary material

Supplementary Figure 1

Supplementary Table 1
