# Supplementary figures and images for: Time to progression ratio in cancer patients enrolled in early phase clinical trials: time for new guidelines?
Source: Br J Cancer. 2018 Oct 17;119(8):937–9. doi: 10.1038/s41416-018-0245-0 (PMC6203755; doi:10.1038/s41416-018-0245-0)

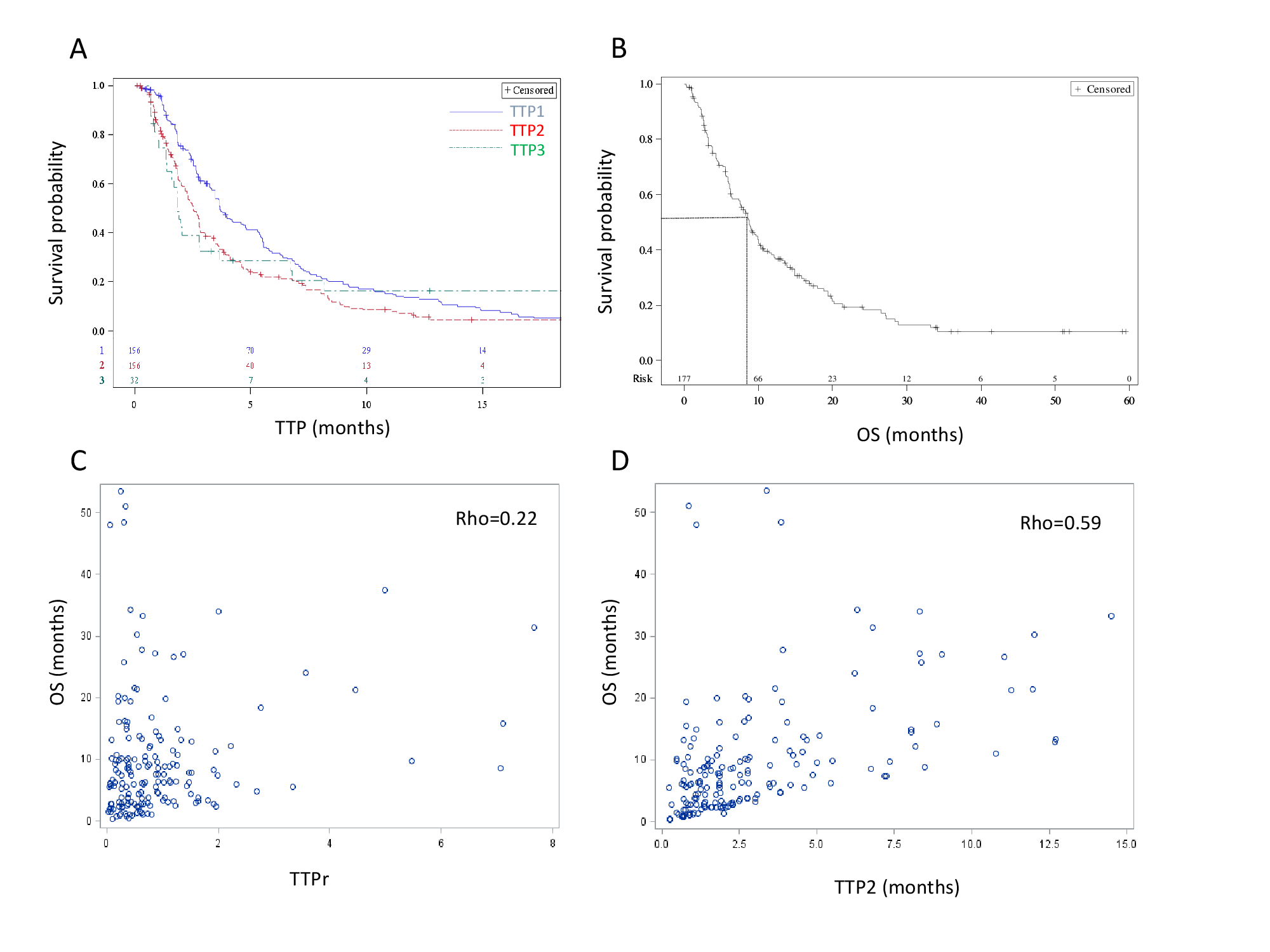

Supplement: Supplementary file 1 — Supplementary Figure 1 [file 41416_2018_245_MOESM1_ESM.tif]
