# Supplementary material for: Time to progression ratio in cancer patients enrolled in early phase clinical trials: time for new guidelines?
Source: Br J Cancer. 2018 Oct 17;119(8):937–9. doi: 10.1038/s41416-018-0245-0 (PMC6203755; doi:10.1038/s41416-018-0245-0)
Supplement: Supplementary file 2 — Supplementary Table 1 [file 41416_2018_245_MOESM2_ESM.docx]

| **Patients** | **Number**  **(N=177)** | **Frequency**  **(%)** |
| --- | --- | --- |
| **Gender**  *Male*  *Female* | 80  97 | 45.2  54.8 |
| **Age at inclusion (years)**  *Mean (Range)* | 55 (21-84) | - |
| **Time since diagnosis (years)**  *Mean (Range)* | 4 (0-28) | - |
| **Primary tumor site**  *Gastrointestinal*  *Breast*  *Head and Neck*  *Lung*  *Prostate*  *Urological tract*  *Skin*  *Gynecological tract*  *Sarcomas*  *Lymphomas*  *Other* | 32  16  9  30  12  8  13  13  8  30  6 | 18.1  9.0  5.1  16.9  6.8  4.5  7.4  7.4  4.5  16.9  3.4 |
| **Prognostic score ***  *0*  *1*  *2* | 93  63  21 | 52.5  35.6  11.9 |
| **Number of previous treatment lines**  *0*  *1-2*  *3-5*  *>5* | 10  72  75  20 | 5.6  40.7  42.4  11.3 |
| **Molecular therapeutic orientation****  **YES**  *- 1st trial only*  *- 2^nd^ trial only*  -*At least two trials*  **NO** | 22  25  9  121 | 12.4  14.1  5.1  68.4 |
| **Trials** | **Number** | **Frequency**  **(%)** |
| **Trial Phase**  **(N=101 trials)**  *Phase 1*  *Phase 2* | 95  6 | 94.1  5.9 |
| **Trial Treatment**  **(N=101 trials)**  *Monotherapy*  *Combination* | 56  45 | 55.4  44.6 |
| **Molecularly enriched trial**  **(N=101 trials)**  *YES (target detail)*  -*CEA*  -*ALK*  -*BRAF*  -*EGFR*  -*FGFR*  -*IDH*  -*MET*  -*PI3K*  *NO* | 20  3  3  4  2  4  2  1  1  81 | 19.8  80.2 |
| **Therapeutic class**  **(N=159 therapeutic agents)**  *Cytotoxic chemotherapy*  *Kinase inhibitor*  *Immune checkpoint blocker*  *Monoclonal antibody*  *Epigenetic targeted therapy*  *Other* | 23  56  16  34  12  18 | 14.5  35.2  10.1  21.4  7.5  11.3 |
| **Allocated doses**  **(N=387 lines of treatment)**  *<25% RP2D*  *25-50% RP2D*  *50-75% RP2D*  *75-125% RP2D*  *>125% RP2D*  *not available* | 42  56  49  207  15  18 | 10.8  14.5  12.7  53.5  3.9  4.6 |

**Supplementary Table 1 : Patients’ and trials’ characteristics**

* RMH score for solid tumors based on LDH, albumin, and number of metastatic sites (range 0-3); Composite score for lymphomas based on LDH and albumin (range 0-2).

** Patients for which at least one trial was based on the detection of a molecular alteration resulting in the inclusion in a dedicated trial (i.e precision medicine).

*** The standardized allocated dose was calculated as the percentage of the maximum tolerated dose (MTD) or maximum administered dose (if no MTD was identified).

Abbreviations: ALK: anaplastic lymphoma kinase; CEA: CarcinoEmbrynonic Antigen; EGFR: Epidermal Growth Factor Receptor; FGFR: Fibroblast Growth Factor Receptor; IDH: Isocitrate dehydrogenase: PI3K: Phosphatidyl-inositol 3 kinase; RP2D: recommended phase 2 dose
